# Obesity is associated with myelin oligodendrocyte glycoprotein antibody-associated disease in acute optic neuritis

**DOI:** 10.1038/s41598-022-21592-8

**Published:** 2022-12-09

**Authors:** Hadas Stiebel-Kalish, Kerstin Rubarth, Karny Shouchane-Blum, Alon Tiosano, Itay Lotan, Mark A. Hellmann, Adi Wilf-Yarkoni, Omer Bialer, Eoin P. Flanagan, Sean J. Pittock, M. Tariq Bhatti, Tanja Schmitz-Hübsch, Friedemann Paul, Susanna Asseyer, John J. Chen

**Affiliations:** 1 Sackler Faculty of Medicine, Tel Aviv University, Tel Aviv, Israel; 2 Felsenstein Medical Research Center, Petach Tikva, Israel; 3 Division of Neuro-Ophthalmology, Department of Neuro-Ophthalmology, Rabin Medical Center, Beilinson Hospital, 4941492 Petach Tikva, Israel; 4 Institute of Biometry and Clinical Epidemiology, Charité-Universitätsmedizin Berlin, Corporate Member of Freie Universität Berlin, Humboldt Universität zu Berlin, Berlin, Germany; 5 Berlin Institute of Health (BIH), Berlin, Germany; 6 Institute of Medical Informatics, Charité-Universitätsmedizin Berlin, Corporate Member of Freie Universität Berlin, Humboldt Universität zu Berlin, Berlin, Germany; 7 Department of Neurology, Rabin Medical Center, Beilinson Hospital, Petach Tikva, Israel; 8 Departments of Ophthalmology and Neurology, Mayo Clinic, Rochester, MN USA; 9 Department of Neurology, Laboratory Medicine and Pathology, Center for MS and Autoimmune Neurology, Mayo Clinic, Rochester, MN USA; 10 Experimental and Clinical Research Center, Max Delbrueck Center for Molecular Medicine, Charité-Universitätsmedizin Berlin, Corporate Member of Freie Universität Berlin, Humboldt-Universität zu Berlin, Berlin Institute of Health, Berlin, Germany; 11 Present Address: The Permanente Medical Group, Oakland, NC USA

**Keywords:** Neurology, Risk factors, Neurological disorders, Multiple sclerosis

## Abstract

Optic neuritis (ON) is a frequent presentation at onset of multiple sclerosis (MS), neuromyelitis optica spectrum disorder (NMOSD), and myelin oligodendrocyte glycoprotein antibody-associated disease (MOGAD). The pathophysiology underlying these diseases, especially MOGAD, is still being elucidated. While obesity has been reported to potentially be a risk factor for MS, this has not been explored in NMOSD or MOGAD. We aimed to investigate a possible association between obesity (body mass index [BMI] > 30 kg/m^2^) in patients with MOGAD, aquaporin 4-IgG positive NMOSD (AQP4-IgG+ NMOSD) or MS. In this multicenter non-interventional retrospective study, data was collected from patients with a first ever demyelinating attack of ON subsequently diagnosed with MOGAD (n = 44), AQP4-IgG+ NMOSD (n = 49) or MS (n = 90) between 2005 and 2020. The following data was collected: age, sex, ethnicity, BMI (documented before corticosteroid treatment), and the ON etiology after diagnostic work-up. A mixed model analysis was performed to assess the potential of obesity or BMI to predict MOGAD-ON, and to distinguish MOGAD-ON from AQP4-IgG+ NMOSD-ON and MS-ON. Main outcome measures included BMI in patients with acute ON and subsequent diagnosis of MOGAD, AQP4-IgG+ NMOSD or MS. A higher BMI was significantly associated with a diagnosis of MOGAD-ON (*p* < 0.001); in MOGAD patients the mean BMI was 31.6 kg/m^2^ (standard deviation (SD) 7.2), while the mean BMI was 24.7 kg/m^2^ (SD 5.3) in AQP4-IgG+ NMOSD patients, and 26.9 kg/m^2^ (SD 6.2) in MS patients. Mixed-effects multinomial logistic regression, adjusted for age and sex, with obesity as a binary variable, revealed that obesity was associated with a higher odds ratio (OR) of a subsequent MOGAD diagnosis (OR 5.466, 95% CI [2.039, 14.650], *p* = 0.001) in contradistinction with AQP4-IgG+ NMOSD. This study suggests an association between obesity and MOGAD. Our findings require further exploration, but could have significant pathophysiologic implications if confirmed in larger prospective studies.

## Introduction

Optic neuritis (ON) is one of the most common clinical presentations at disease onset of myelin oligodendrocyte glycoprotein antibody-associated disease (MOGAD), neuromyelitis optica spectrum disorder (NMOSD), and multiple sclerosis (MS)^1,2^. The pathophysiology of these diseases is still being elucidated, especially for MOGAD because it is the most recently described entity^3,4^. Prior studies have suggested that obesity may play a predisposing risk factor for MS^5–7^, but this has not been explored in aquaporin 4-IgG positive NMOSD (AQP4-IgG+ NMOSD) or MOGAD.

MOGAD-ON can sometimes be mistaken for pseudotumor cerebri because patients can present with severe headaches and bilateral optic disc edema^8–10^. We serendipitously observed that another common similarity to pseudotumor cerebri is that many MOGAD patients have a high body mass index (BMI) at disease onset. Therefore, the goal of this study was to investigate the hypothesized association between BMI and MOGAD-ON compared to AQP4-IgG+ NMOSD-ON and MS-ON.

## Patients and methods

**Figure 1:**
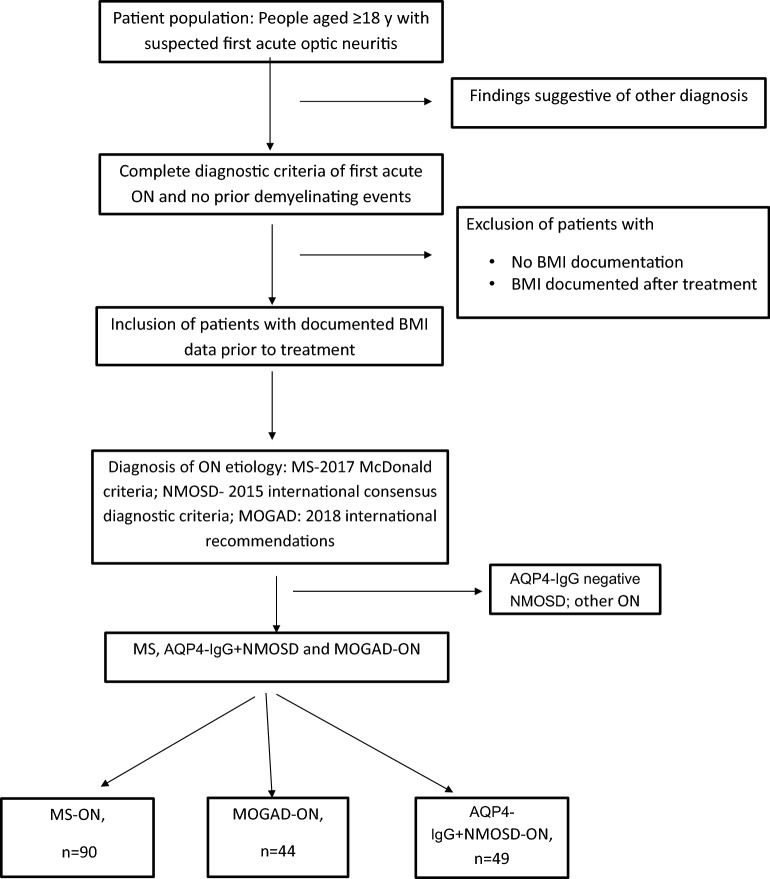
Patient selection and study design flowchart.

The study was conducted in accordance with the Declaration of Helsinki. Following institutional review board approval (Israel: RMC-0498-18; USA: 21-001492, Germany: EA1/182/10), data was collected from adult patients (age ≥ 18 years) presenting with first-ever ON in one of three teaching hospitals (Rabin Medical Center, Israel; Mayo Clinic, Rochester, MN, USA; Charité-Universitätsmedizin Berlin, Germany) between 2005 and 2020, and who were subsequently diagnosed with MOGAD, AQP4-IgG+ NMOSD or MS. Since only patients with a first episode of ON and with no prior clinical demyelination attack were included, no bias from corticosteroid-induced weight gain, other immunotherapy-induced weight-effects, or bias from possible weight loss in chronic MS, was introduced. Additionally, patients for whom BMI data was not documented at presentation and before ON treatment, were excluded (Fig. 1).

ON was diagnosed based on a combination of at least three of the following clinical findings: decreased visual acuity, pain with eye movement, visual field defect, a relative afferent pupillary defect, changes in color vision, optic disc swelling on fundus examination, and/or compatible magnetic resonance imaging (MRI) findings. Diagnosis of the underlying etiology was performed as part of the clinical routine and documented at follow-up visit respecting diagnostic criteria for MS according to the 2017 McDonald criteria^11^, NMOSD according to the 2015 international consensus diagnostic criteria and positive serum AQP4-IgG by cell-based assay^12^, and MOGAD at presentation in patients with clinical characteristics consistent with MOGAD-ON and positive serum MOG-IgG^13^. Serological testing for MOG-IgG was conducted at presentation in all optic neuritis patients presenting with findings suggestive of MOGAD, as proposed by Jarius et al. in the international recommendations on diagnosis of MOGAD^14^. Testing for MOG-IgG in all MS patients is not warranted, as the positive predictive value for MOG-IgG is only 72%, and routine testing may lead to a significant increase in false positives^15,16^. Patients with AQP4-IgG+ NMOSD were not necessarily tested for MOG-IgG due to the rare coexistence of both antibodies^14^.

Up until 2018, serological testing for MOG-IgG was carried out using live cell-based assays at the Institut d’Investigacio Biomedica August Pi I Sunyer, Barcelona, Spain (for patients from Israel) or at the Mayo Clinic Neuroimmunology laboratory, Rochester, MN, USA. Patients from Berlin were only included after 2018. Since 2018, commercial cell-based assays (Euroimmun AG Lübeck, Germany) were used in Germany and Israel, while patients from the USA continued to be tested using live cell-based assays at the Mayo Clinic Neuroimmunology laboratory. Patients with seronegative NMOSD were excluded.

Overweight and obesity was defined as abnormal or excessive fat accumulation that presents a risk to health. Using the World Health Organization's criteria, this study defined a BMI over 25 kg/m^2^ as overweight, and over 30 kg/m^2^ as obese (https://www.who.int/news-room/fact-sheets/detail/obesity-and-overweight). Age, sex, ethnicity, and BMI were recorded for each patient prior to beginning treatment with corticosteroids and/or other immunotherapy.

### Statistical analysis

Demographics were compared between groups with parametric Analysis of Variance (ANOVA) in case of metric variables and chi-squared test in case of categorical variables. To evaluate the BMI differences between the three diseases, a multinomial logistic regression adjusted for BMI, age, and sex, including a random effect for study center, was performed. To explore whether obesity was associated with MOGAD, and to obtain odds ratios, a mixed-model analysis with obesity as a binary variable was performed, again adjusted for age and sex as potential confounders. The reference category for disease was set to AQP4-IgG+ NMOSD. To compare the effects of BMI between study centers, separate multinominal logistic regression models were fitted.

Due to the small sample size of the study center in Germany, this was only done with the data from Israel and the USA. After analyzing the results from Israel, the analysis was then independently repeated in the cohorts from Germany and the USA. The finding of higher obesity rates in MOGAD was replicated despite these other centers not knowing the results of the Israeli cohort until after their results were submitted.

A *p*-value < 0.05 was considered to be statistically significant. Due to the exploratory character of the study, no correction for multiplicity on p-values was applied. Therefore, the results should be considered exploratory, non-confirmatory. Statistical analyses were performed using SPSS (IBM Statistics Software, IBM Corporation Version 27, 2021) and R Studio (R Project for Statistical Computing, Version 4.1.0).

### Ethical approval

Institutional review board approval (Israel: RMC-0498-18; USA: 21-001492, Germany: EA1/182/10) with waiver of patient consent in anonymous retrospective de-identified data collection for Israel and USA, and with written consent for Germany (available upon request).

## Results

183 patients were included in this study. The overall mean age at time of ON was 39 years (standard deviation (SD) 14.09), 36.6% were male, and the mean BMI was 27.43 kg/m^2^ (SD 6.67). In Israel, 25% (21/84) of the patients were classified as obese, which is comparable to the 27% (3/11) of patients in Germany. The proportion of obese patients in the USA was 34% (30/88). Subsequent etiology for ON was AQP4-IgG+ NMOSD in 49 patients (26.8%), MOGAD in 44 patients (24.0%), and MS in 90 patients (49.2%). Table 1 details the mean age, sex, and BMI of patients, stratified by ON etiology. After analyzing the results from Israel, the analysis was then independently repeated in the cohorts from Germany and the USA. The pooled and site-specific results are detailed in Table 1.

**Table 1:** Demographic characteristics of patients with first-ever acute optic neuritis (ON) stratified by etiology.

	MOGAD n = 44	AQP4-IgG+ NMOSD n = 49	MS n = 90	*p*-value
Age at ON onset (years), mean (SD)/median [IQR]	44.48 (14.20)/45.50 [32.50, 54.00]	41.84 (16.92)/38.00 [26.00, 54.00]	34.54 (10.72)/32.00 [25.25, 42.00]	< 0.001
Female sex, n (%)	27 (61%)	42 (86%)	27 (52%)	< 0.001
BMI (kg/m^2^), mean (SD)	31.60 (7.15)	24.69 (5.31)	26.89 (6.18)	< 0.001
Overweight, n (%)	12 (27.27%)	8 (16.33%)	23 (25.56%)	0.387
Obese, n (%)	23 (52.27%)	8 (16.33%)	23 (25.56%)	< 0.001

MOGAD, myelin oligodendrocyte glycoprotein antibody-associated disease; AQP4-IgG+ NMOSD, aquaporin 4-IgG positive neuromyelitis optica spectrum disorder; MS, multiple sclerosis; ON, optic neuritis; SD, standard deviation; IQR, interquartile range; N, sample size; BMI, body mass index.

**Figure 2:**
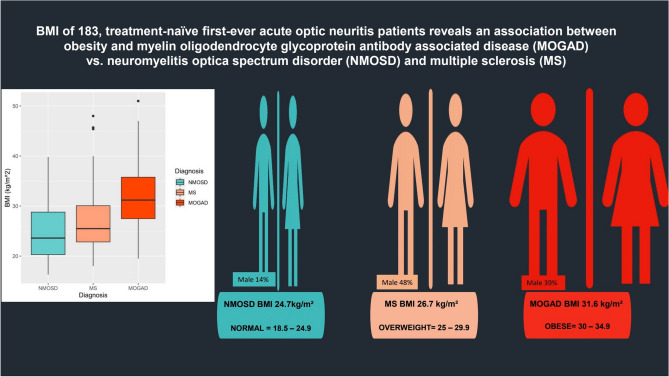
Infographic of body mass index (BMI) at presentation of first-ever acute optic neuritis in patients with myelin oligodendrocyte glycoprotein antibody-associated disease (MOGAD), neuromyelitis optica spectrum disorder (NMOSD), and multiple sclerosis (MS). Legends: Plots demonstrating the distribution of BMI by group. The boxplot lines correspond to the 25th, 50th and 75th percentiles. The male symbol represents the percentage of male patients in each group.

Mean (SD) age was 41.84 years (17.0) in AQP4-IgG+ NMOSD, 44.84 years (14.2) in MOGAD, and 34.54 years (10.7) in MS (p < 0.001). The mean (SD) BMI was 31.6 kg/m^2^ (7.2) in the MOGAD group, 26.9 kg/m^2^ (6.2) in the MS group, and 24.7 kg/m^2^ (5.3) in the AQP4-IgG+ NMOSD group (*p* < 0.001), as depicted in Fig. 2.

**Figure 3:**
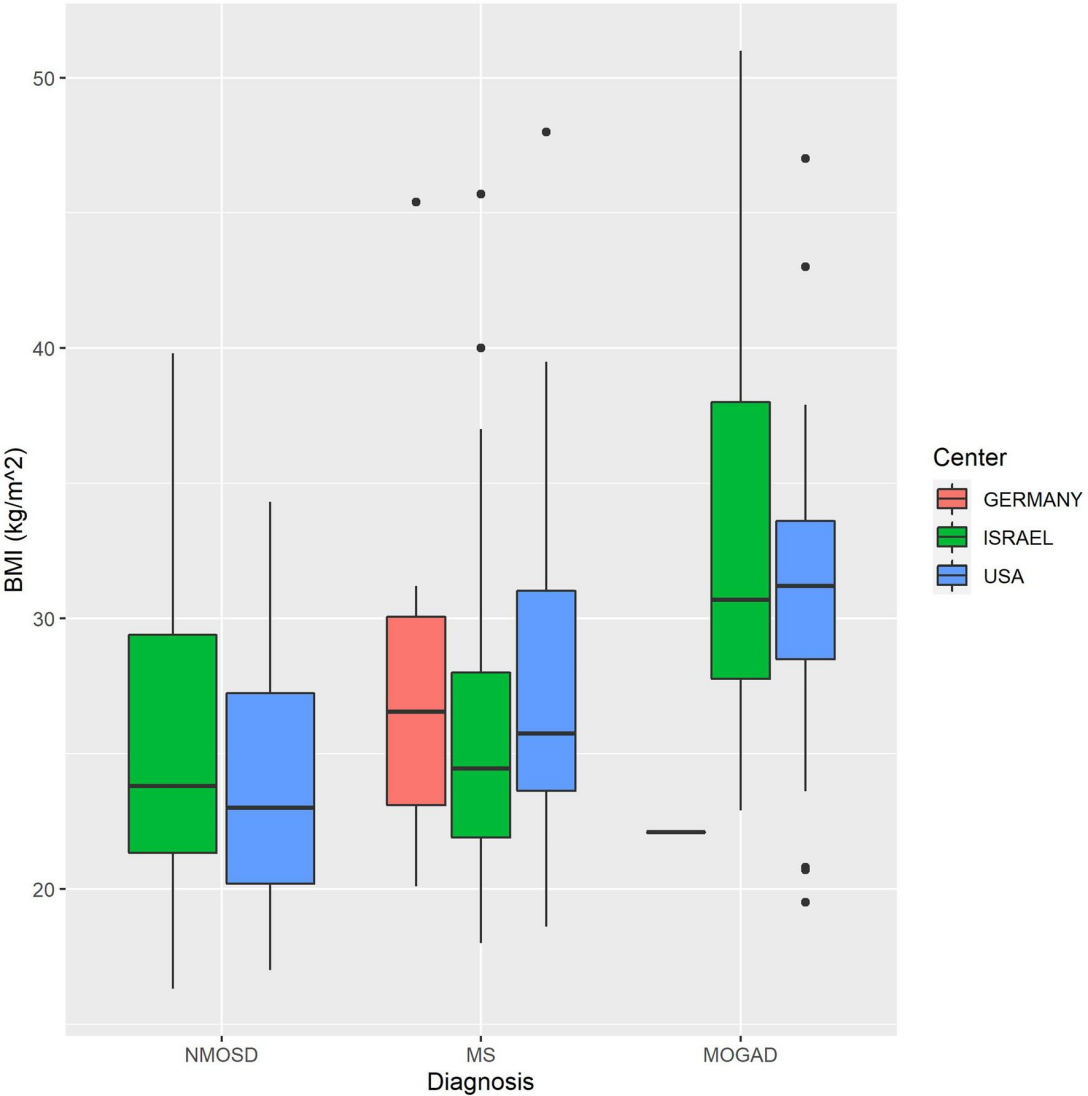
Body mass index (BMI) at presentation of 183 patients with first-ever acute optic neuritis is similar within the respective disease categories across the cohorts from Israel, the USA and Germany. Legends: Plots demonstrating the distribution of BMI by group. The boxplot lines correspond to the 25th, 50th and 75th percentiles.

Figure 3 depicts the means of BMI in the three cohorts from Israel, the USA and Germany.

All patients were White, except for one MOGAD patient (BMI of 33.5) and three AQP4-IgG+ NMOSD patients (mean BMI 24.7 kg/m2) who were Black (mean BMI 24.7 kg/m^2^, one AQP4-IgG+ NMOSD patient who was Asian (BMI 19.6 kg/m^2^), one AQP4-IgG+ NMOSD patient who was American Indian (BMI 26.6 kg/m^2^), and one AQP4-IgG+ NMOSD patient who was Hispanic (BMI 29.0 kg/m^2^).

### Mixed-effects multinomial logistic regression

In patients with acute ON, obesity was associated with a higher odds ratio (OR) of subsequent MOGAD diagnosis (OR 5.466, 95% CI [2.039, 14.650], *p* = 0.001) compared with AQP4-IgG+ NMOSD (Supplementary Table 1). A higher BMI showed a trend towards a subsequent MS diagnosis following ON (OR 1.1074, 95% CI [0.998, 1.115], *p* = 0.056), (Fig. 2, Supplementary Tables 1, 2).

The mixed-model analysis, adjusted for sex, age and BMI, for obtaining the diagnosis MOGAD or MS in reference to AQP4-IgG+ NMOSD revealed that the OR for females to be diagnosed as MOGAD and MS was 0.31 (95% confidence interval (CI): [0.106, 0.911], *p* = 0.033) and 0.172 (95% CI [0.066, 0.447], *p* < 0.001), respectively, in comparison to being diagnosed with AQP4-IgG+ NMOSD. Increasing age was negatively associated with the diagnosis of MS in comparison with AQP4-IgG+ NMOSD (OR: 0.959, 95% CI [0.931, 0.988], *p* = 0.005).

The ethnic variance between the centers, and within each diagnosis group, as detailed in the paragraph above, was too low to add ethnicity to the mixed-effects model analysis.

The detailed results are shown in Supplementary Tables 3 and 4. The OR for BMI in the mixed-effects multinomial logistic regression models are 1.190 (MOGAD vs. AQP4-IgG+ NMOSD) and 1.074 (MS vs. AQP4-IgG+ NMOSD), and are comparable with the fixed-effects multinomial logistic regression models using Israel data (OR: 1.182, 1.021 respectively) and USA data (OR:1.281, 1.165 respectively) only.

## Discussion

Our study found an association between obesity (BMI ≥ 30 kg/m^2^) and the likelihood of a MOGAD diagnosis in patients with acute ON. While obesity during adolescence has been reported to be associated with an increased risk of MS^17^, to our knowledge, the association between MOGAD and obesity has not been explored in the past**.** Obesity is a well-known risk factor in immunological diseases^18,19^. Potential mechanisms include that levels of vitamin D metabolites are lower in obese people than in people with normal weight, and that decreased levels of serum 25-hydroxyvitamin D appear to increase MS risk. Furthermore, adipose tissue produces a variety of proinflammatory cytokines, including leptin. Leptin induces proinflammatory Th1-regulated immune responses and reduces regulatory T-cell activity^20^. Th1-promoting effects of obesity may increase the risk of developing MS, in particular in subjects with a HLA-genetic susceptibility to both MS and obesity^21^.

In our cohort, patients with MS-ON had a higher BMI compared to patients with a AQP4-IgG+ NMOSD-related ON. While obesity was associated with a diagnosis of MOGAD, there was only a trend toward obesity among MS patients. This may suggest a relationship between obesity-induced inflammation and a higher risk for MOGAD. If this finding is corroborated in future larger studies and in varying ethnicities, it may uncover a novel pathogenic mechanism in MOGAD. In addition, a heightened awareness of a possible correlation between obesity and MOGAD may prevent the misdiagnosis of pseudotumor cerebri in obese patients with severe headaches and bilateral disc edema.

Limitations of this study include its retrospective nature, and that data was recorded primarily from ON cases from three neuroimmunology tertiary referral centers, which may have caused a bias towards more atypical or severe disease. In addition, patients were predominantly White, and, therefore, our conclusions may not be applicable to other ethnicities. There was not enough ethnicity variance among the centers to include it in the mixed-model analysis, but the few Asian, American Indian, Black, and Hispanic patients included in the study did not differ from the average BMI within the respective disease category. BMI data was collected only from patients presenting with ON because they were mostly recruited from neuro-ophthalmology clinics. It is expected, however, that the differences in BMI would extend across other presentations, e.g. myelitis. A study comprising a larger number of patients with different presentations are required to confirm these findings. Finally, since this was a hypothesis-generating study, we did not assess comorbidities, lipid profiles and medications used. These may be potential confounders which will need to be addressed in future studies. The main strength of our study is that it results from real-life clinical practice and objectivizes a finding from clinical observation within a multicenter study.

## Conclusions

Our study suggests that there is an association between obesity and MOGAD in a retrospective study of 183 patients: 44 with MOGAD, 49 with AQP4-IgG+ NMOSD, and 90 with MS. A higher BMI was significantly associated with a diagnosis of MOGAD (*p* < 0.001); in MOGAD patients the mean BMI was 31.6 kg/m^2^ (standard deviation (SD) 7.2), while the mean BMI was 24.7 kg/m^2^ (SD 5.3) in AQP4-IgG+ NMOSD patients, and 26.9 kg/m^2^ (SD 6.2) in MS patients. Future prospective investigation of the association between obesity and MOGAD may help shed light on the pathogenic mechanisms of this intriguing disorder.

## Supplementary Information

Supplementary Information.

## Data Availability

Data are available upon reasonable request.

## References

[1] Jitprapaikulsan J, Chen JJ, Flanagan EP (2018). Aquaporin-4 and myelin oligodendrocyte glycoprotein autoantibody status predict outcome of recurrent optic neuritis. Ophthalmology.

[2] Jurynczyk M, Messina S, Woodhall MR (2017). Clinical presentation and prognosis in MOG-antibody disease: a UK study. Brain.

[3] Spadaro M, Winklmeier S, Beltrán E (2018). Pathogenicity of human antibodies against myelin oligodendrocyte glycoprotein. Ann. Neurol..

[4] Dale RC, Tantsis EM, Merheb V (2014). Antibodies to MOG have a demyelination phenotype and affect oligodendrocyte cytoskeleton. Neurol. Neuroimmunol. NeuroInflamm..

[5] Mokry LE, Ross S, Timpson NJ, Sawcer S, Davey Smith G, Richards JB (2016). Obesity and multiple sclerosis: a mendelian randomization study. PLoS Med..

[6] Guerrero-García JDJ, Carrera-Quintanar L, López-Roa RI, Márquez-Aguirre AL, Rojas-Mayorquín AE, Ortuño-Sahagún D (2016). Multiple sclerosis and obesity: possible roles of adipokines. Mediators Inflamm..

[7] Gianfrancesco M, Barcellos L (2016). Obesity and multiple sclerosis susceptibility: a review. J. Neurol. Neuromed..

[8] Lotan I, Brody J, Hellmann MA (2018). Myelin oligodendrocyte glycoprotein-positive optic neuritis masquerading as pseudotumor cerebri at presentation. J. Neurol..

[9] Narayan RN, Wang C, Sguigna P, Husari K, Greenberg B (2019). Atypical anti-MOG syndrome with aseptic meningoencephalitis and pseudotumor cerebri-like presentations. Mult. Scler. Relat. Disord..

[10] Biotti D, Bonneville F, Tournaire E (2017). Optic neuritis in patients with anti-MOG antibodies spectrum disorder: MRI and clinical features from a large multicentric cohort in France. J. Neurol..

[11] Thompson AJ, Banwell BL, Barkhof F (2018). Diagnosis of multiple sclerosis : 2017 revisions of the McDonald criteria. Lancet Neurol..

[12] Wingerchuk DM, Banwell B, Bennett JL (2015). International consensus diagnostic criteria for neuromyelitis optica spectrum disorders. Neurology.

[13] Marignier R, Hacohen Y, Cobo-Calvo A (2021). Myelin-oligodendrocyte glycoprotein antibody-associated disease. Lancet Neurol.

[14] Jarius S, Paul F, Aktas O (2018). MOG encephalomyelitis : international recommendations on diagnosis and antibody testing. J. Neuroinflamm..

[15] Manzano GS, Salky R, Mateen FJ (2022). Positive predictive value of MOG-IgG for clinically defined MOG-AD within a real-world cohort. Front. Neurol..

[16] Sechi E, Buciuc M, Pittock SJ (2021). Positive predictive value of myelin oligodendrocyte glycoprotein autoantibody testing. JAMA Neurol..

[17] McKay KA, Jahanfar S, Duggan T, Tkachuk S, Tremlett H (2017). Factors associated with onset, relapses or progression in multiple sclerosis: a systematic review. Neurotoxicology.

[18] Tsigalou C, Vallianou N, Dalamaga M (2020). Autoantibody production in obesity: is there evidence for a link between obesity and autoimmunity?. Curr. Obes. Rep..

[19] Versini M, Jeandel PY, Rosenthal E, Shoenfeld Y (2014). Obesity in autoimmune diseases: not a passive bystander. Autoimmun. Rev..

[20] Hedström AK, Olsson T, Alfredsson L (2012). High body mass index before age 20 is associated with increased risk for multiple sclerosis in both men and women. Mult. Scler. J..

[21] Hedström AK, Lima Bomfim I, Barcellos L (2014). Interaction between adolescent obesity and HLA risk genes in the etiology of multiple sclerosis. Neurology.

