# Author Correction: Obesity is associated with myelin oligodendrocyte glycoprotein antibody-associated disease in acute optic neuritis

**DOI:** 10.1038/s41598-023-30115-y

**Published:** 2023-02-20

**Authors:** Hadas Stiebel-Kalish, Kerstin Rubarth, Karny Shouchane-Blum, Alon Tiosano, Itay Lotan, Mark A. Hellmann, Adi Wilf-Yarkoni, Omer Bialer, Eoin P. Flanagan, Sean J. Pittock, M. Tariq Bhatti, Tanja Schmitz-Hübsch, Friedemann Paul, Susanna Asseyer, John J. Chen

**Affiliations:** 1 Sackler Faculty of Medicine, Tel Aviv University, Tel Aviv, Israel; 2 Felsenstein Medical Research Center, Petach Tikva, Israel; 3 Division of Neuro-Ophthalmology, Department of Neuro-Ophthalmology, Rabin Medical Center, Beilinson Hospital, 4941492 Petach Tikva, Israel; 4 Institute of Biometry and Clinical Epidemiology, Charité-Universitätsmedizin Berlin, Corporate Member of Freie Universität Berlin, Humboldt Universität zu Berlin, Berlin, Germany; 5 Berlin Institute of Health (BIH), Berlin, Germany; 6 Institute of Medical Informatics, Charité-Universitätsmedizin Berlin, Corporate Member of Freie Universität Berlin, Humboldt Universität zu Berlin, Berlin, Germany; 7 Department of Neurology, Rabin Medical Center, Beilinson Hospital, Petach Tikva, Israel; 8 Departments of Ophthalmology and Neurology, Mayo Clinic, Rochester, MN USA; 9 Department of Neurology, Laboratory Medicine and Pathology, Center for MS and Autoimmune Neurology, Mayo Clinic, Rochester, MN USA; 10 Experimental and Clinical Research Center, Max Delbrueck Center for Molecular Medicine, Charité-Universitätsmedizin Berlin, Corporate Member of Freie Universität Berlin, Humboldt-Universität zu Berlin, Berlin Institute of Health, Berlin, Germany; 11 Present Address: The Permanente Medical Group, Oakland, NC USA

Correction to: *Scientific Reports* https://doi.org/10.1038/s41598-022-21592-8, published online 09 December 2022

The original version of this Article contained an error in the name of the author Friedemann Paul, which was incorrectly given as Paul Friedemann.

The original Article has been corrected.

