# Supplementary material for: Obesity is associated with myelin oligodendrocyte glycoprotein antibody-associated disease in acute optic neuritis
Source: Sci Rep. 2022 Dec 9;12:21312. doi: 10.1038/s41598-022-21592-8 (PMC9734097; doi:10.1038/s41598-022-21592-8)
Supplement: Supplementary file 1 — Supplementary Information. [file 41598_2022_21592_MOESM1_ESM.docx]

Supplemental files:

# Supplemental Table 1: Fixed coefficients, p-values, Odds Ratios and their 95% confidence intervals obtained from mixed effects multinomial logistic regression (reference diagnosis: NMOSD) including obesity as a binary predictor

| Diagnosis | Variable | Coefficient | p-value | Odds Ratio | 95%-CI(Odds Ratio) |
| --- | --- | --- | --- | --- | --- |
| MOGAD | Intercept | -0.234 | 0.778 | 0.792 | [0.155, 4.053] |
|  | Gender = Female | -1.275 | **0.018** | 0.280 | [0.098, 0.799] |
|  | Age | 0.012 | 0.430 | 1.012 | [0.982, 1.043] |
|  | Obesity | 1.699 | **0.001** | 5.466 | [2.039, 14.650] |
| MS | Intercept | 3.537 | **<0.001** | 34.364 | [6.363, 185.571] |
|  | Gender = Female | -1.821 | **<0.001** | 0.162 | [0.063, 0.415] |
|  | Age | -0.38 | **0.009** | 0.962 | [0.935, 0.991] |
|  | Obesity | 0.560 | 0.249 | 1.751 | [0.673, 4.553] |

Abbreviations: myelin oligodendrocyte glycoprotein antibody disease (MOGAD); neuromyelitis optica spectrum disorder (NMOSD); multiple sclerosis (MS); BMI (body mass index).

# Supplemental Table 2: Fixed coefficients, p-values, exponentiated coefficients and their 95% confidence intervals obtained from mixed effects multinomial logistic regression (reference diagnosis: NMOSD) including BMI in kg/m^2 as a continuous predictor

| Diagnosis | Variable | Coefficient | p-value | Odds Ratio | 95%-CI(Odds Ratio) |
| --- | --- | --- | --- | --- | --- |
| MOGAD | Intercept | -4.470 | 0.002 | 0.011 | [0.001, 0.180] |
|  | Gender = Female | -1.169 | **0.033** | 0.311 | [0.106, 0.911] |
|  | Age | 0.009 | 0.582 | 1.009 | [0.977, 1.041] |
|  | BMI | 0.174 | **<0.001** | 1.190 | [1.099, 1.289] |
| MS | Intercept | 1.928 | 0.122 | 6.879 | [0.594, 79.691] |
|  | Gender = Female | -1.761 | **<0.001** | 0.172 | [0.066, 0.447] |
|  | Age | -0.420 | **0.005** | 0.959 | [0.931, 0.988] |
|  | BMI | 0.071 | 0.056 | 1.074 | [0.998, 1.155] |

Abbreviations: myelin oligodendrocyte glycoprotein antibody disease (MOGAD); neuromyelitis optica spectrum disorder (NMOSD); multiple sclerosis (MS); BMI (body mass index).

# Supplemental Table 3: Coefficients, p-values, exponentiated coefficients (Odds Ratios) and their 95% confidence intervals obtained from multinomial logistic regression from Israel data (reference diagnosis: NMOSD) including BMI in kg/m^2 as a continuous predictor

| Diagnosis | Variable | Coefficient | p-value | Exp(Coefficient) | 95%-CI(Coefficient) |
| --- | --- | --- | --- | --- | --- |
| MOGAD | Intercept | -4.210 | **0.034** | 0.015 | [0.000, 0.722] |
|  | Gender = Female | -1.314 | 0.116 | 0.269 | [0.052, 1.382] |
|  | Age | -0.008 | 0.787 | 0.992 | [0.938, 1.050] |
|  | BMI | 0.167 | **0.003** | 1.182 | [1.058, 1.321] |
| MS | Intercept | 2.457 | 0.102 | 11.672 | [0.616, 221.321] |
|  | Gender = Female | -1.057 | 0.103 | 0.348 | [0.098, 1.239] |
|  | Age | -0.048 | **0.027** | 0.953 | [0.914, 0.994] |
|  | BMI | 0.021 | 0.656 | 1.021 | [0.931, 1.120] |

Abbreviations: myelin oligodendrocyte glycoprotein antibody disease (MOGAD); neuromyelitis optica spectrum disorder (NMOSD); multiple sclerosis (MS); BMI (body mass index).

# Supplemental Table 4: Coefficients, p-values, exponentiated coefficients (Odds Ratios) and their 95% confidence intervals obtained from multinomial logistic regression from USA data (reference diagnosis: NMOSD) including BMI in kg/m^2 as a continuous predictor

| Diagnosis | Variable | Coefficient | p-value | Exp(Coefficient) | 95%-CI(Coefficient) |
| --- | --- | --- | --- | --- | --- |
| MOGAD | Intercept | -6.024 | **0.004** | 0.002 | [0.000, 0.000] |
|  | Gender = Female | -1.480 | 0.069 | 0.228 | [0.046. 1.123] |
|  | Age | -0.035 | 0.509 | 1.015 | [0.971. 1.061] |
|  | BMI | 0.248 | **<0.001** | 1.281 | [1.122, 1.462] |
| MS | Intercept | -0.227 | 0.905 | 0.797 | [0.019, 33.052] |
|  | Gender = Female | -2.760 | **<0.001** | 0.063 | [0.014. 0.282] |
|  | Age | -0.035 | 0.118 | 0.965 | [0.923, 1.009] |
|  | BMI | 0.152 | **0.021** | 1.165 | [1.023, 1.326] |

Abbreviations: myelin oligodendrocyte glycoprotein antibody disease (MOGAD); neuromyelitis optica spectrum disorder (NMOSD); multiple sclerosis (MS); BMI (body mass index).
